# Dietary Intake of Individual (Intrinsic and Added) Sugars and Food Sources from Spanish Children Aged One to <10 Years—Results from the EsNuPI Study †

**DOI:** 10.3390/nu14081667

**Published:** 2022-04-16

**Authors:** Marina Redruello-Requejo, María de Lourdes Samaniego-Vaesken, Teresa Partearroyo, Paula Rodríguez-Alonso, María José Soto-Méndez, Ángela Hernández-Ruiz, Federico Lara Villoslada, Rosaura Leis, Emilio Martínez de Victoria, José Manuel Moreno, Rosa M. Ortega, María Dolores Ruiz-López, Ángel Gil, Gregorio Varela-Moreiras

**Affiliations:** 1 Grupo USP-CEU de Excelencia “Nutrición para la vida (Nutrition for life)”, ref: E02/0720, Departamento de Ciencias Farmacéuticas y de la Salud, Facultad de Farmacia, Universidad San Pablo-CEU, CEU Universities, Urbanización Montepríncipe, 28660 Boadilla del Monte, Spain; m.redruello@usp.ceu.es (M.R.-R.); l.samaniego@ceu.es (M.d.L.S.-V.); t.partearroyo@ceu.es (T.P.); 2 Spanish Nutrition Foundation (FEN), c/General Álvarez de Castro 20, 1apta, 28010 Madrid, Spain; prodriguez@fen.org.es (P.R.-A.); mariarosaura.leis@usc.es (R.L.); 3 Iberoamerican Nutrition Foundation (FINUT), Av. del Conocimiento 12, 3a pta, Armilla, 18016 Granada, Spain; msoto@finut.org (M.J.S.-M.); ahernandez@finut.org (Á.H.-R.); emiliom@ugr.es (E.M.d.V.); mdruiz@ugr.es (M.D.R.-L.); agil@ugr.es (Á.G.); 4 Instituto de Nutrición Puleva, Camino de Purchil 66, 18004 Granada, Spain; federico.lara@lactalis.es; 5 Department of Pediatrics, Unit of Pediatric Gastroenterology, Hepatology and Nutrition, University Clinical Hospital of Santiago, IDIS, ISCIII, University of Santiago de Compostela, 15700 Santiago de Compostela, Spain; 6 CIBEROBN (Physiopathology of Obesity and Nutrition CB12/03/30038), Instituto de Salud Carlos III (ISCIII), 28029 Madrid, Spain; 7 Department of Physiology, Faculty of Pharmacy, Campus de Cartuja, University of Granada, s/n, 18071 Granada, Spain; 8 Pediatric Department, Calle Marquesado de Sta. Marta 1, University of Navarra Clinic, 28027 Madrid, Spain; jmoreno@unav.es; 9 Department of Nutrition and Food Science, Faculty of Pharmacy, Complutense University of Madrid, Plaza Ramón y Cajal s/n, 28040 Madrid, Spain; rortega@ucm.es; 10 Department of Nutrition and Food Science, Faculty of Pharmacy, Campus de Cartuja, s.n, University of Granada, 18071 Granada, Spain; 11 Institute of Nutrition and Food Technology “José Mataix,” Biomedical Research Center, University of Granada, Parque Tecnológico de la Salud, Avenida del Conocimiento s/n, Armilla, 18100 Granada, Spain; 12 Department of Biochemistry and Molecular Biology II, University of Granada, Campus de Cartuja, s.n, 18071 Granada, Spain

**Keywords:** EsNuPI study, pediatrics, Spanish children, feeding behavior, dietary habits, nutrition assessment, pediatric nutrition, intrinsic sugar, added sugar, free sugars

## Abstract

Currently, in Spain there are no studies assessing the intakes and sources of intrinsic and added sugars by both children consuming standard milks and children regularly consuming adapted milk formulas. Our goal was to evaluate current sugar intake levels (intrinsic and added) and their major dietary sources within the EsNuPI study participants by applying two 24-h dietary recalls that were completed by 1448 children (1 to <10 years) divided into two subsamples: One “Spanish Reference Sample” (SRS) of the general population (*n* = 707) and another sample which included children consuming adapted milks including follow-on milk, toddler’s or growing up milk and fortified and enriched milks, here called “Adapted Milk Consumers Sample” (AMS) (*n* = 741). Estimates of intrinsic and added sugar intakes from the Spanish EsNuPI population as well as the adherence to recommendations varied notably according to age segment, but no major differences between subsamples were found. Younger children (1 to <3 years) showed the highest added sugar contribution to total energy intake (TEI) (SRS: 12.5% for boys and 11.7% for girls; AMS: 12.2% for boys and 11.3% for girls) and the lowest adherence to recommendations set at <10% TEI (SRS: 27.4% for boys and 37.2% for girls; AMS: 31.3% for boys and 34.7% for girls). Adherence increased with age but remains inadequate, with approximately one in two children from the older age segment (6 to <10 years) exceeding the recommendations. Main food sources of intrinsic sugars for both subsamples were milk and dairy products, fruits, vegetables and cereals, while for added sugars, these were milk and dairy products (mainly yogurts), sugars and sweets (mainly sugary cocoa and nougat), bakery products (mainly cookies) and cereals (mainly bread and wheat flour). However, for the AMS, the groups milk and dairy products and cereals showed a significantly lower contribution to intrinsic sugar intake but a significantly higher contribution to that of added sugars. These results demonstrate that sugar intake and the adherence to recommendations in the studied population varied notably according to age but not to the type of milk consumed. In addition, our results highlight the need to monitor the consumption of added sugars by the infant population, as well as the need to make efforts to facilitate this task, such as harmonizing the recommendations regarding free/added sugars and the inclusion of information on their content on the nutritional labeling of products in order to incorporate them into food composition databases.

## 1. Introduction

The global burden of the overweight and obesity pandemic, predominantly amongst children, is also prevalent in Spain from an early age. The 2019 report from the ALADINO study (Diet, Physical Activity, Child Development and Obesity in Spain) [1], performed with 16,665 schoolchildren between six and nine years old, revealed that 40.6% had excess weight, of which 23.3% were overweight and 17.3% presented obesity. Furthermore, the study describes that there was a higher number of obese children amongst those belonging to lower-income families or a socially vulnerable background [1].

Childhood obesity has a deep negative impact on health, both during childhood and in adulthood, and constitutes in itself a risk factor for being an adult with obesity and developing further chronic diseases; in fact, for this reason it has been regarded as a “crisis in public health” [2]. Therefore, children are an especially vulnerable target population group and there is an urgent need to develop and establish convenient public health strategies to tackle this multifactorial problem [3]. Amongst other factors, sedentarism and unbalanced, low-quality “westernized” diets play a key role in the development of this condition.

Excessive sugar consumption, mainly added sugar through sugar-sweetened beverages, has been related to an increase in overall energy intake and a reduction of the intake of more nutritionally dense foods, leading to an unhealthy diet and weight gain, dental caries decay and higher risk of non-communicable diseases [4]. The World Health Organization (WHO) established a recommendation to reduce the intake of free sugars throughout the life course both in adults and children and a strong recommendation to reduce the intake of free sugars to less than 10% of total energy intake (TEI) [5]. The WHO guidelines also suggested a further reduction of the intake of free sugars to below 5% of TEI as a conditional recommendation to achieve potential additional health benefits [5]. Moreover, several institutions such as the ESPGHAN (European Society for Pediatric Gastroenterology, Hepatology and Nutrition) suggest that added sugar intake should be even lower in infants and children below two years of age [6].

Nevertheless, inconsistencies in the definition of “sugars” from different official bodies complicate the interpretation of their recommendations and the comparison across surveys. On one hand, sugars can be categorized as “intrinsic”, i.e., those that are naturally present in the structure or matrix of whole fresh fruits and vegetables and milk and dairy products without further processing; and on the other, as “extrinsic” or “added”. According to the European Food Safety Authority (EFSA), added sugars comprise “sucrose, fructose, glucose, starch hydrolysates (glucose syrup, high-fructose syrup) and other isolated sugar preparations used as such or added during food preparation and manufacturing” [7]. Conversely, the term “free sugars”, according to the WHO, refers to added sugars plus sugars naturally present in honey, syrups, fruit juices and fruit juice concentrates [8]. Official recommendations focus on extrinsic sugars because there is no sound evidence of adverse effects of an excessive intake of intrinsic sugars [5]. While the terms “added” and “free” are not interchangeable, the limit of a maximum intake of 10% of TEI is common to other guidelines that prefer to address their recommendation in reference to added sugars—as these are the ones that can be acted upon to effectively reduce intake—such as the Dietary Guidelines for Americans 2020–2025 established by the U.S. Department of Agriculture (USDA) [9]. In Europe, the EFSA does indicate that a reduction in sugar consumption should be recommended due to the high frequency of sugar intake in foods and beverages and its potential to increase the risk of dental caries, although for the time being there is insufficient data to establish a maximum limit for sugar intake, particularly for added sugars [7]. Nonetheless, some European countries have issued national recommendations regarding sugar intake. UK’s SACN (Scientific Advisory Committee on Nutrition) have set out recommendations addressing free sugar intake, which should account for no more than 5% of TEI [8]. In Spain, the Spanish Agency for Food Safety and Nutrition (AESAN) has endorsed the WHO recommendations set at 10% of TEI for free sugars [10] However, it also issued recommendations for added sugar intake at less than 30 g/day, referring to those sugars “taken separately or used as ingredients in processed or prepared foods (e.g., white sugar, brown sugar, unrefined sugar, corn syrups, malt syrup, maple syrup, fructose sweeteners, liquid fructose, honey, molasses, anhydrous dextrose and crystallized dextrose)” [11].

In this regard, a recent position paper on the definition of added sugars and their declaration on the labeling of food products in Spain, signed by 145 Spanish scientists in the field of nutrition, states that it is necessary and urgent to be able to unify the criteria for the use of sugars as an ingredient and nutritional information, particularly in the form of added sugars. One of the reasons why this position paper recommended the use of the added sugars criterion is that it also advocates for the declaration of added sugars in the nutritional information on the labeling of food products, not only to know their quantity, but also as a valid tool for intake assessments [12]. So far, only a few countries have regulated the mandatory addition of this declaration on the labelling, such as the US [13] and Mexico [14], which have also preferred to refer to the added sugar content in the carbohydrate section, after that of total sugars. In Spain (as well as in the rest of Europe) added sugar contents are not required by law to be declared on food labels; therefore, the assessment and monitorization of their contents and intakes becomes a difficult task.

According to the EFSA Panel on Nutrition, Novel Foods and Food Allergens (NDA) [15], there is a great variability in added and free sugar intake across Europe, but main food groups contributing to the intakes of both types of sugars are common to all: Sugar and confectionery (table sugar, honey, syrups, confectionery and water-based sweet desserts), beverages (sugar-sweetened soft and fruit drinks, fruit juices) and bakery. As observed, added and free sugars mainly originate from non-core food groups, but the EFSA acknowledges an exception for milk and dairy products in young consumers, where in infants, children and adolescents, sweetened milk and dairy products are also major contributors to mean intakes of added and free sugars [15]. This is one of the reasons why in the present study we wanted to pay special attention to dairy consumption at early ages.

In Spain, data from the ANIBES study (anthropometric data, macronutrients and micronutrients intake, practice of physical activity, socioeconomic data, and lifestyles in Spain), a cross-sectional study of a nationally representative sample of the Spanish population (from 9 to 75 years old) carried out in 2013 [16], showed a total sugar intake of 91.6 g/day for children aged 9–12 years (*n* = 213), of which 48.6 g/day (9.8% TEI) were added sugars. Interestingly, added sugar consumption was significantly higher in children and adolescents, with up to two-fold differences in added sugar contribution to TEI when compared to older groups. As a result, only 58.2% of children met the current WHO recommendations (<10% TEI), dropping to 9.4% when considering the conditional ones (<5% TEI). Sugar sweetened soft drinks, bakery and pastries were the major food sources of added sugars, although other food products such as chocolates, yogurts, fermented milks and other dairy products, juices and nectars, breakfast cereals and cereal bars also had a significant contribution [16]. The ENALIA study [17], performed on Spanish children and adolescents aged 6 months to 17 years, showed similar results with a total sugar intake of 95.1 g/day. Specifically, added sugar intake in this population group was 48 g/day (10.4% TEI). Data from different subgroups showed that children from 3 to 9 years old were those with the greater energy levels obtained from added sugars with an average intake of 52.7 g/day, (11.7% TEI). These data showed that more than half of the studied population exceeded the limit of 10% of TEI established by the WHO [17].

Strategies to promote reductions of sugar intake, including the reformulation of relevant dietary sugar sources such as beverages, breakfast cereals and bakery goods, have been established by Spanish authorities in arrangement with the food industry, as described by the “Collaboration Plan for the improvement of the composition of food and beverages” [18], which envisioned a progressive reduction of around 10% of median added sugar content by 2020. So far, the direct impact of the Plan on sugar intakes of the Spanish population has not been assessed. Nonetheless, it is worth noting that this initiative was implemented on added sugars because, as already mentioned, these are the ones that are easiest to act upon. It is for this reason that the authors have preferred to focus our efforts on assessing the consumption of added sugars, abiding by the aforementioned definition of the AESAN [11], which is consistently in line with the definition provided by the EFSA [19].

In the present work derived from the EsNuPI study (“Nutritional Study in Spanish Pediatric Population”), our aim was to assess current sugar intake levels (intrinsic and added) and their major dietary sources from Spanish children aged one to <10 years old. Two population groups were compared, one consuming adapted milk formulas and another that only consumed regular milk, in order to evaluate whether their dietary patterns differ with respect to intrinsic and added sugar intakes and their main food sources.

## 2. Materials and Methods

### 2.1. Study Design and Sample

The EsNuPI study comprises a prospective, cross-sectional, observational study, conducted between October 2018 and January 2019. The comprehensive design, protocol and methodology have been published elsewhere [20]. The EsNuPI study assessed the dietary patterns and nutrient intake, in addition to physical activity and sedentary behaviors of Spanish children living in urban areas with >50,000 inhabitants, from nine geographical areas, as established by Nielsen. Two subsamples were compared, aged one to <10 years old, one including the urban non-vegan individuals who only consumed natural, standard milk in the last 12 months, the “Spanish Reference Sample, SRS”, and another called “Adapted Milk Consumers Sample, AMS” of non-vegan subjects from urban areas who only consumed adapted milks over the last 12 months. The “adapted milks” denomination included infant formulas, follow-on milk formulas, toddler’s milk formulas (also called “young children milk formula” and in Spain “growing up” milk formula) and fortified and enriched milk formulas (i.e., with added docosahexaenoic acid (DHA), calcium, vitamin D, iron). The originally estimated sample included 1500 individuals, and the sample errors were ±2.52% and ±2.59%, respectively, for a 95.5% confidence level and estimation of equally probable categories (*p* = *q* = 50%), considering a universe of 2,205,646 children. A total of 1514 children (*n* = 742 SRS; *n* = 772 AMS) were recruited and finally 1448 individuals completed the study (95.6% response rate). The EsNuPI study was completed in accordance with the declaration of Helsinki, approved by the University of Granada ethical committee (No. 659/CEIH/2018) and registered in ClinicalTrials.gov (Unique Protocol ID: FF01/2019).

### 2.2. Procedures and Data Collection

Participants completed a face-to-face interview providing sociodemographic information and a first 24-h dietary recall (24-h DR). After seven days, a second 24-h DR was answered by participants by telephone.

#### Socio-Demographic and Anthropometric Information

In the first interview, a general questionnaire was used to collect the following variables: Place and date of birth, gender, academic level of parents or caregivers (elementary or less/secondary/university/higher education), place of residence, family income level, lifestyle, activity patterns and sedentary behaviors. Height and weight data were declared by parents or caregivers, based on the child’s pediatric health card. Body mass index (BMI; kg/m^2^) was calculated as weight (kg) divided by squared height (m). Spanish BMI cut-off criteria were used to define underweight, overweight or obesity: “underweight” (percentile 4 for boys and 10 for girls), “overweight” (percentile 79 for boys and 89 for girls) and “obesity” (percentile 97.5 for boys and 99 for girls) [21].

### 2.3. Procedures, Dietary Survey and Data Collection

Parents or caregivers enabled the completion of two independent 24-h DRs to determine children’s dietary intake (proxy report), which included a week and a weekend day (non-consecutive), one being face-to-face and the other by telephone ≥7 days later. A detailed description of dietary intake including ingredients and methods of preparation was organized as mealtimes (breakfast, mid-morning, lunch, mid-afternoon, dinner and other moments) to calculate energy and nutrient distribution during the day. The study of food and nutrient consumption involves many methodological difficulties, especially when it comes to assessing it in children. One of them is calculation of misreporting, which was performed by the protocol proposed by the EFSA, based on the work by Goldberg [22] and Black [23] that evaluates the reported energy intake (EIrep) against the presumed energy requirements. The results included in the present manuscript were not adjusted for misreporting because in the previous work from Madrigal et al. [24] the exclusion of misreporters resulted in no significant differences in the total energy intake (TEI) and the distributions of relative macronutrient intakes. Hence, it was assumed that it does not significantly modify the results and conclusions of this study.

Resources such as the “Tables of common home measures and habitual portion sizes for Spain population” [25] and the “Photo guide of common portions sizes of Spanish foods” [26] were used by interviewers in order to facilitate the adequate completion of the survey. In addition, the “VD-FEN 2.1” software, a Dietary Evaluation Program developed by the Spanish Nutrition Foundation (FEN), was used to calculate the food, beverage and energy and nutrient intakes.

### 2.4. Quantification of Sugar Consumption

After energy and nutrient calculation, the proportion of “intrinsic” and “added” sugars were calculated through food product labeling according to their brand, and considering their total sugar contents obtained from the Food Composition Tables (FCTs) by Moreiras et al. [27].

#### 2.4.1. Selection of Food Products and Brands

For each coded food and beverage declared by participants from the EsNuPI Study (a total of 746 from which 327 were fresh with no available label), full labeling of packaged products was collected in order to be representative of at least >80% of the Spanish market, as a weighted average by sales. Photographs were taken in retail centers (hypermarkets, supermarkets and convenience stores) from two to seven food products either from traditional manufacturer’s brands or from distribution brands (supermarket’s own), comprising a total of 1164 food products. For each, up to four different photographs were taken with the aim of obtaining precise information about packaging, company, brand, nutritional labeling and ingredient lists (3037 photographs). An additional online survey was also undertaken to find those products that were not found in the field work (less than 10%).

#### 2.4.2. Classification and Quantification of Sugars in Food

Foods and beverages with “intrinsic” sugars included all fresh and unprocessed foods which did not carry a label and those without any added ingredients: Most fresh fruits, vegetables, meats, fish, etc., as well as those packaged/labelled foods without any kind of added sugars indicated in the ingredients list.

“Added sugars” were defined in accordance to the Regulation of the European Union 1924/2006 on nutrition and health claims made on foods [19] and Regulation 1169/2011 on the provision of food information to consumers [28], as: “any added mono- or disaccharides or any other food used for its sweetening properties”.

Foods with added sugars were all those packaged/labelled foods for which the ingredients list indicated some form of “free sugars” such as: Sugar, honey, caramel (E-150), glucose, fructose, sucrose, lactose, dextrose, syrup (any of the above) and any of the above inverted or partially inverted.

For the calculation of the intrinsic and added sugar contents the following criteria were considered:

- Foods without added sugars: It was considered that 100% of the total sugars listed in the FCT are intrinsic.
- Foods with added sugars: In cases where the percentage of added sugars was declared on the ingredient list of the nutritional labelling, the amount of added sugars was directly calculated and the amount of intrinsic sugars was estimated by difference with the total sugars content declared. In those cases where the percentage of added sugars was not declared, the intrinsic sugars content was calculated based on the content of each of the ingredients in the product, using the nutritional composition obtained from the FCT [27]. The above referred amount was subtracted from the total sugars content of the nutritional product labeling to estimate the added sugar content. Next, the weight percentages for both types of sugars were estimated, so that X% INTRINSIC and X% ADDED were applied to the total sugars of the FCT of each coded food.
  - Subgroup of milks (natural, standard milks and adapted milk formulas): Given the focus of this article on this food subgroup, the intrinsic and added sugars provided by these foods were calculated directly with the % of intrinsic and added sugars estimated for the specific type and brand of milk consumed by each participant.
  - The rest of food the groups apart from the milk subgroup: The intake of intrinsic and added sugars provided by these foods was calculated from the average % of intrinsic and added sugars calculated for the brands according to their proportion of consumption.
- Foods considered properly as added sugar: Due to their nutritional characteristics and according to current legislation, white sugar, brown sugar and honey were considered 100% added sugars.

### 2.5. Statistical Analysis

A total of 746 food items resulting from the collection of reported dietary intake data were categorized into 18 food groups (see Supplementary Material Table S1) as previously described [20] and further transformed into energy and nutrient data for analysis. All estimates were made from an average across the two days of diet recording.

The Kolmogorov–Smirnoff normality test was used to determine the normality of the distribution of the variables. Median and interquartile range (IQR) were used for continuous variables and frequencies and percentages for categorical variables to describe intrinsic and added sugar intake by type of sample (SRS and AMS), gender and age groups.

Comparisons between SRS and AMS by gender and age group were completed by the Mann–Whitney U-test. The Kruskal–Wallis test and the Dunn method to adjust for multiple comparison and adjustment of the *p* value with Bonferroni correction were used to calculate differences among each age group within samples. Level of significance was established at *p* < 0.05. Data analyses were performed using IBM SPSS 27.0 (IBM Corp., Armonk, NY, USA).

## 3. Results

### 3.1. Usual Intake of Intrinsic and Added Sugars in Children

Intrinsic and added sugar intakes are described in Table 1 for each sample, segmented by gender and age. When comparing samples, we observed significant differences for intrinsic sugar intakes amongst girls aged 1 to <3 years, where the AMS had lower intrinsic sugar consumption (*p* ≤ 0.05). Likewise, significant differences were observed for added sugar consumption between girls aged 1 to <3 years, where higher intakes were observed amongst those from the SRS (*p* ≤ 0.05).

Amongst each sample, when results were analyzed by age group we observed that added sugar intakes were significantly higher (*p* ≤ 0.05) amongst older groups only in the AMS, independently of gender.

Added sugar distributions as a percentage of total energy intake (TEI) are shown in Table 2. Girls aged 6 to <10 years from SRS showed significantly lower TEI from added sugars (9.4%) than those who consumed adapted milk (11.0%) (*p* ≤ 0.05). On the other hand, it is worth mentioning that, regardless of the type of milk consumed, boys and girls from early ages (1 to <3 years) had a higher added sugar contribution to TEI than those from older age segments.

We also assessed the percentage of children who met the WHO guidelines for sugar intake [5], which strongly recommend the decrease of free sugar consumption throughout the lifecycle to less than 10% of TEI (Table 3). In addition, these include a consideration that a further reduction to less than 5% TEI would have additional health benefits (Table 4). In this regard, we found that in all groups less than 50% of individuals met the recommendations by WHO, except for girls aged 6 to <10 years from the SRS in which 58.0% complied. In addition, it is worth underlining that girls aged 6 to <10 years from the SRS had a higher level of compliance with recommendations than those consuming adapted milk (58.0% and 39.5%, respectively).

Table 5 details the intake of added sugars according to body composition, a criterion for which no significant differences were found within each sample. When comparing the two samples we only observed one significant difference: Among underweight children, those aged 3–6 years from the AMS consumed more added sugars than those in the SRS (*p* ≤ 0.05).

Intakes of added sugars segmented by socio-economic level are presented in Table 6. Again, no significant differences were found within each sample. Among samples, the intakes of added sugars in the income range 2001–3000 € for children aged 3–6 years were significantly higher from the AMS (40.6 g/day) with respect to the SRS (31.0 g/day) (*p* ≤ 0.05).

### 3.2. Contribution of Food and Beverage Groups to Reported Intakes of Intrinsic and Added Sugar 

The main sources of intrinsic and added sugar intakes amongst children from the EsNuPI study are presented in the following figures. It is worth noting that in all figures, data are presented as the median of proportional contribution and only for those foods which contributed at least 1% to intrinsic or added sugar intakes of the population.

**Figure 1:**
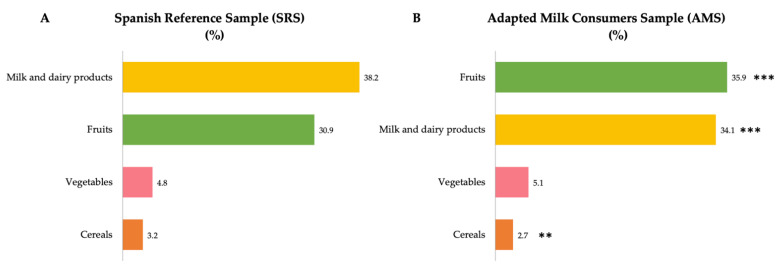
Dietary food and beverage groups contributing to intrinsic sugar intakes (%) from the EsNuPI study population (“Spanish Pediatric Population”) in both the Spanish Reference Sample (**A**) and the Adapted Milk Consumers Sample (**B**). ** *p* ≤ 0.01 compared to reference sample (Mann–Whitney test); *** *p* ≤ 0.001 compared to reference sample (Mann–Whitney test). Only foods contributing ≥1% to total intrinsic sugar intakes of the population have been included.

**Figure 2:**
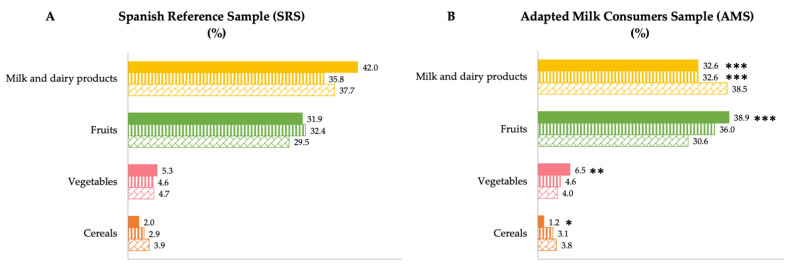
Dietary food and beverage groups contributing to intrinsic sugar intakes (%) from the EsNuPI study population (“Spanish Pediatric Population”) in both the Spanish Reference Sample (**A**) and the Adapted Milk Consumers Sample (**B**) by age group. * *p* ≤ 0.05 compared to reference sample (Mann–Whitney test); ** *p* ≤ 0.01 compared to reference sample (Mann–Whitney test); *** *p* ≤ 0.001 compared to reference sample (Mann–Whitney test). Only foods contributing ≥1% to total intrinsic sugar intakes of the population have been included.

Figure 1 shows the median contribution (%) of main food sources to intrinsic sugar intakes. Specifically, food groups with the highest median proportional contribution to total intrinsic sugar intake in the reference sample (SRS) were firstly milk and dairy products (38.2%), followed by fruits (30.9%). Thirdly, vegetables accounted for 4.8% of total intrinsic sugar intakes and, finally, the cereals contributed 3.2% to the total intrinsic sugar intake (Figure 1A). Conversely, we found that amongst the sample of adapted milk consumers (AMS), the largest contributors to intrinsic sugar intakes were fruits (35.9%), closely followed by milk and dairy products (34.1%), then vegetables (5.1%) and cereals (2.7%) (Figure 1B). In turn, we observed that milk and dairy products contributed in a lower proportion to total intrinsic sugar intakes from children in the AMS than in the SRS (*p* ≤ 0.001) (Figure 1). However, fruits contributed in a higher proportion to total intrinsic sugar intakes from children in the AMS than in the SRS (*p* ≤ 0.001). In addition, cereals accounted for a lower proportion when compared to intrinsic sugar intakes from children of the SRS (Figure 2).

Figure 2 shows the median contribution (%) of food groups to intrinsic sugar intake, categorized by age group for both samples. For children in the AMS aged 1 to 3 years, significantly different contributions were observed, being higher for the fruit (*p* ≤ 0.001) and vegetable (*p* ≤ 0.01) groups and lower for the milk and derivatives (*p* ≤ 0.001) and cereals (*p* ≤ 0.05) groups compared to SRS children of the same age segment. However, at older ages these differences disappear and only a lower contribution to intrinsic sugars from the milk and derivatives group is observed in children from 3 to <6 years of the AMS compared to the SRS (*p* ≤ 0.001).

**Figure 3:**
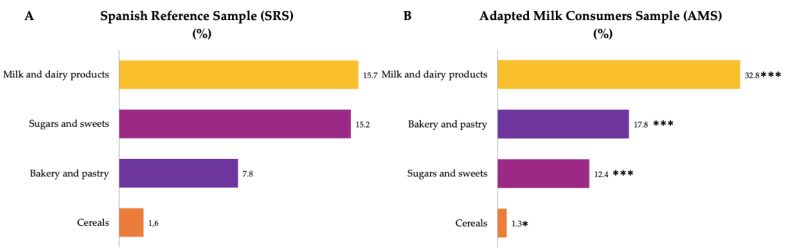
Dietary food and beverage groups contributing to added sugar intakes (%) from the EsNuPI study population (“Spanish Pediatric Population”) in both the Spanish Reference Sample (**A**) and the Adapted Milk Consumers Sample (**B**). * *p* ≤ 0.05 compared to reference sample (Mann–Whitney test); *** *p* ≤ 0.001 compared to reference sample (Mann–Whitney test). Only foods contributing ≥1% to total added sugar intakes of the population have been included.

Food groups with the highest median contribution (%) to added sugar intakes were milk and dairy products, sugars and sweets, bakery and pastry and cereals (Figure 3) for both groups. The contribution of milk and dairy products, bakery and pastry and sugars and sweets were significantly higher amongst children of the AMS than in the SRS.

**Figure 4:**
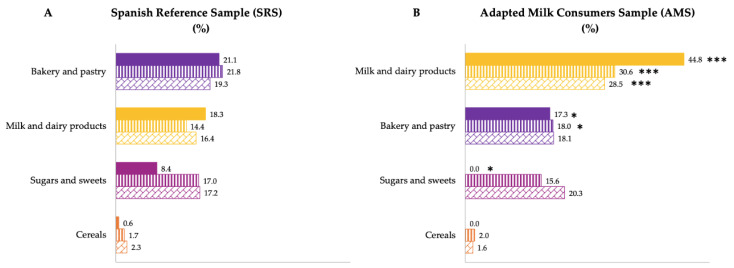
Dietary food and beverage groups contributing to added sugar intakes (%) from the EsNuPI study population (“Spanish Pediatric Population”) in both the Spanish Reference Sample (**A**) and the Adapted Milk Consumers Sample (**B**) by age-group. * *p* ≤ 0.05 compared to reference sample (Mann–Whitney test); *** *p* ≤ 0.001 compared to reference sample (Mann–Whitney test). Only foods contributing ≥1% to total added sugar intakes of the population have been included.

Additionally, Figure 4 shows the median contribution (%) of food groups to added sugar intake, categorized by age group for both samples. There were differences by groups of age between both samples (Figure 4). AMS children from 1 to 3 years of age had a higher contribution of added sugars from milk and dairy products (*p* ≤ 0.001) and a lower added sugar contribution from bakery and pastry (*p* ≤ 0.05) and sugars and sweets (*p* ≤ 0.05) than SRS children of the same age. Likewise, AMS children from 3 to 6 years of age had a higher contribution of added sugars from milk and dairy products (*p* ≤ 0.001) and a lower added contribution from bakery and pastry (*p* ≤ 0.05) than SRS children of the same age. Finally, the older age group of the AMS had a higher contribution of this nutrient from the milk and dairy products (*p* ≤ 0.001) than their counterparts of the SRS.

**Figure 5:**
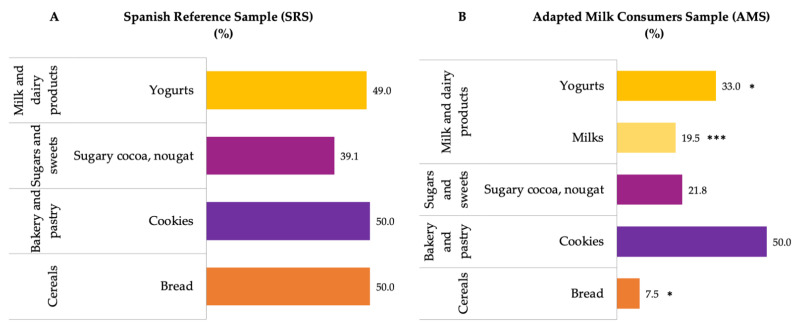
Bread and wheat flour, cookies, sugary cocoa, nougat, yogurts and milks subgroups contributing to added sugar intakes in the cereals, bakery and pastry, sugars and sweets, and milk and dairy products groups (%) from the EsNuPI study population (“Spanish Pediatric Population”) in both the Spanish Reference Sample (**A**) and the Adapted Milk Consumers Sample (**B**). * *p* ≤ 0.05 compared to reference sample (Mann–Whitney test). *** *p* ≤ 0.001 compared to reference sample (Mann–Whitney test).

Examining the food subgroups that were identified as the major sources of added sugars in both samples, among milk and dairy products, the yogurt subgroup had the highest median proportional contribution to total added sugar intake, being significantly higher in the SRS than in the AMS (*p* ≤ 0.001) (Figure 5). However, the contribution of cereals was significantly lower amongst the AMS than in the SRS (*p* ≤ 0.05, respectively). Likewise, it is interesting to underline that the subgroups of bread and wheat flour, cookies, sweetened cocoa and nougats were those that contributed most to the intake of added sugars in the groups of cereals, bakery and pastries, and sugars and sweets, respectively.

**Figure 6:**
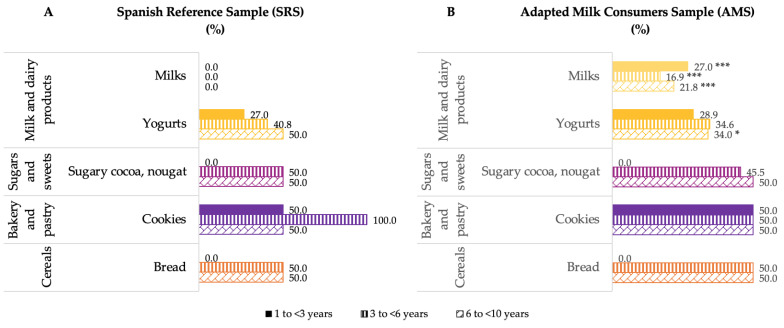
Bread and wheat flour, cookies, sugary cocoa, nougat, yogurts and milks subgroups contributing to added sugar intakes in cereals, bakery and pastry, sugars and sweets, and milk and dairy products groups (%) from the EsNuPI study population (“Spanish Pediatric Population”) in both the Spanish Reference Sample (**A**) and the Adapted Milk Consumers Sample (**B**) by age-group. * *p* ≤ 0.05 compared to reference sample (Mann–Whitney test). *** *p* ≤ 0.001 compared to reference sample (Mann–Whitney test).

When comparing samples by age group (Figure 6), SRS children from 1 to <3 years and 3 to <6 years had a higher contribution to added sugar intake from the milk subgroup than their counterparts of the AMS (*p* ≤ 0.001). However, AMS children between 6 and <10 years had a higher contribution to added sugar intake from the yogurts (*p* ≤ 0.05) and milks (*p* ≤ 0.001) subgroups than SRS children of the same age.

## 4. Discussion

### 4.1. Usual Intrinsic and Added Sugar Intake in Children

The EsNuPI study encompassed a representative sample of the Spanish children population in the age range of 1 to <10 years old and it is the first study in our country to evaluate and compare dietary patterns and food sources between two samples of milk consumers. Our results provide an update on the dietary intake of a population group that is critical for the prevention of diet-related diseases and new and relevant information on the differences between children who consume adapted milks and those who do not, a topic that lacks scientific evidence.

As expected, a general trend is observed where median daily intake of intrinsic sugars increases with age, although there were no significant differences in sugar intakes (both intrinsic and added) between the two samples. However, for added sugar intake, this increasing trend is only observed for the AMS and not for the SRS, whose intakes seem to remain more stable. Nevertheless, when transforming these intakes into the % of TEI, this trend is inverted, peaking for boys and girls from early ages (1 to <3 years) and decreasing with age, regardless of the type of milk consumed. Consequently, the prevalence of adequacy to the recommendations of % TEI contributed by added sugars [5,9] increases with age, being roughly two times higher in older children (6 to <10 years) than in younger children (1 to <3 years), also for both samples. It should be noted that the girls from the AMS did not show such marked trends, which may be related to the imbalance of the sample size in the 6 to <10 years segment.

The low percentage of prevalence of compliance with recommendations observed in both samples, regardless of gender and age, is remarkable. These results are even more discouraging when considering WHO’s conditional recommendation setting the limit at 5% of TEI, for which a substantial drop is observed as not even 10% of the participants would meet it. All this considering that the present study evaluated the intake of added sugars, and not free sugars, as referred to in the WHO recommendations, which would also include sugars naturally present in natural juices. In this regard, a study by Amoutzopoulos et al. [29] assessing sugar intake in the UK (*n* = 2138) considered in parallel the consumption of added and free sugars, which represented 7% and 13% of TEI, respectively. However, another study in the Portuguese population (*n* = 5811) by Marihno et al. [30] did not observe differences greater than 1% in terms of %TEI contributed by each type of sugar.

The methodological difficulties deriving from the different terminologies used to describe sugars limits not only the ability to compare intakes with recommendations, but also with other studies assessing their intake. For the present EsNuPI study, we chose to consider the added sugars approach rather than the WHO criteria, since the data available for the Spanish population also followed the added sugars criteria. Thus, data from the 2013 ANIBES study [16] for the children population aged 9 to 12 years (*n* = 213) showed mean daily intakes of 48.6 g/day for added sugars, slightly higher than those observed in this study but consistent with the fact that the ANIBES study sample is older. For this reason, it is also consistent that the contribution of added sugars to the percentage of TEI observed for the child population of the ANIBES study was 9.8%, slightly lower. The data observed for the Spanish children population are somewhat worse compared to the data from the aforementioned study in the Portuguese population [30] carried out in 2015–2016, where mean daily intake of added sugars was 28.2 g/day for children <5 years (*n* = 944) and 44.6 g/day for children 5–9 years (*n* = 383), representing 7.6% and 9.6% of TEI, respectively. In the UK, the study by Amoutzopoulos et al. [29] using data from 2014–2016 showed added sugar intakes among children aged 4 to 10 years stood at 38.5 g/day (10% TEI). Data from the IDEFICS Study of 2007–2008 are also available in Europe, where average intake of free sugars (including those from fruit juices) for European children (2–9 years old, *n* = 8308) was 18% of TEI, with a large variation between countries ranging from 13% in Italy to 27% in Germany. Globally, a review from 2016 [31] including nationally representative dietary surveys reported that for young infants, who relied entirely on a milk-based diet, total sugars provided 38% of TEI. This figure decreased to 20–30% of ET when weaning was introduced as older infants began to nurture from a broader variety of sources. Regarding added sugars, intake increased after the age of one year and intakes above 10% TEI have been reported in pre-school children in Australia, the UK and the US (15–17). In school-aged children and adolescents, added sugar intakes are consistently higher than those of younger children, reaching up to 19% TEI. Fortunately, these excessive intakes of added sugars decreased throughout adulthood, remaining below 10% TEI in older adults [31].

Concerning the adherence to the recommendations on limiting the consumption of added sugars to less than 10% of TEI [5,9], the obtained results are again somewhat worse when compared with the studies mentioned above, where adherence reached 58.2% of children aged 9 to 12 years in the ANIBES study [16] and 59.3% of the Portuguese population aged 5 to 9 years, rising to 74.7% for the <5 years age segment [30]. Most discouragingly, in the IDEFICS Study [32], only 19.6% of European children aged 2 to 9 years met the <10% TEI cut-off WHO’s strong recommendation, with only 4.1% meeting the <5% TEI conditional recommendation. In any case, it is necessary to highlight the concerning observation that the percentage of adherence was consistently lower in children and adolescents compared with adults and the elderly, a trend that has also been reported in other studies [17,31,33,34,35] and which is in line with our finding that children of younger ages (1 to <3 years) had a higher contribution of added sugars to TEI than those in older age segments, regardless of their sample group. These observations are worrying because of the metabolic and epigenetic implications of excessive sugar intakes, and it remains unclear if this pattern is due to a generational effect where children are more prone to sweet/sweetened products than adults, or to a trend of increased added sugar intake that might lead children to maintain these high intakes during their lifetime. Nevertheless, the fact that dietary contribution of added sugar is reduced with age could also indicate that healthier dietary patterns are acquired with age and are then transferred into future generations. In this regard, a study in US children aged <2 years old proves that these trends can be reversed, finding that between 2005 and 2016 the consumption of added sugars followed a downward trend [36], which needs to be consolidated in order to reach the WHO targets.

It is a fact that children in low- and middle-income countries are more vulnerable to inadequate prenatal, infant and young child nutrition [37]. Yet at the same time, these children are exposed to foods that are cheap but of poorer nutritional quality: High in fat, sugars and salt, energy-dense and low in micronutrients. It is well known that these dietary patterns are some of the factors responsible for the current childhood obesity pandemic. Despite the fact that apparently the circumstance of living in low- and middle-income countries might be comparable to that of living in low- and middle-income households, in the present study we did not observe significant differences in added sugar intakes with respect to body composition or socio-economic status. Thus, the isolated significant differences we found in this study do not allow us to relate any of the factors considered. One of the reasons why this relationship may have been weak is the fact that in the present study added sugars, rather than free sugars, were quantified. In this respect, ESPGHAN [6] concludes that a higher than recommended intake of free sugars (i.e., mono and disaccharides), particularly from sugar-sweetened beverages, in children and adolescents, is associated with an increased risk of excess weight gain. It has already been mentioned that in the EsNuPi study sample the consumption of these types of beverages was not relevant. However, in Spain, it has already been commented that the results of the ALADINO study (Diet, Physical Activity, Child Development and Obesity in Spain) [1], performed in 2019 in 16,665 schoolchildren between 6 and 9 years, there was a higher number of obese children amongst those belonging to lower-income families or a socially vulnerable background [1]. Indeed, data from the Early Childhood Longitudinal Birth Cohort (ECLS-B) [38], of a nationally representative sample of 14,000 children born in the United States in 2001, show that in kindergarten-age children a combination of socioeconomic factors, including household income as well as parent’s education and occupation, contributed to children’s early obesogenic environment. However, socioeconomic status did not modify other associations found between children’s overweight and obesity and parental smoking, birth weight and not eating dinner as a family [38]. Another study on trends and sociodemographic factors associated with overweight and obesity in Spanish children and adolescents found that overall prevalence of childhood overweight/obesity has slightly decreased during the last decade. However, it has increased among children from the most deprived areas and with non-Spanish nationalities [39].

### 4.2. Contribution of Food and Beverage Groups to Reported Intakes of Intrinsic and Added Sugars 

Analyzing the main food sources observed for each sample, it is worth remarking the significantly lower contribution of the milk and dairy products and cereals groups to the consumption of intrinsic sugars in the AMS, as these two food groups in turn contribute significantly more to the consumption of added sugars by this sample. Nevertheless, the EFSA acknowledges that in infants, children and adolescents, milk and dairy products are core food groups and that therefore sweetened milk and dairy products are also major contributors to mean intakes of added and free sugars in young consumers [15].

Comparison with the data from the Spanish children population aged 9 to 12 years from the ANIBES study [16] showed that the main food sources of both intrinsic and added sugars were common to those described for the EsNuPI child population, although the contribution observed for the milk and dairy products and cereals food groups was more similar to that described here for the SRS. The only relevant differences with respect to findings of the ANIBES study are the contribution of juices and nectars to the intrinsic sugar intake (22.7%) and that of sugar soft drinks (19.7%) to that of added sugars, whose contribution in the present study was less than 1%. This may be explained by the fact that the EsNuPI study population (1 to <10 years) is younger than the ANIBES study population (9 to 12 years). Results from the Portuguese infant population [30] are similar to those of the ANIBES study, but it should be underlined that infant formula was one of the main dietary sources of added sugars in children <5 years, with a contribution of 14.0%. Amongst milk and dairy products, the yogurt subgroup showed the highest contribution to added sugar intakes in both samples. These results are consistent with those observed for children from the ANIBES study where the group yogurts and fermented milks accounted for 8.32% of added sugars, only below the group “other dairy products” that accounted for 9.69% of added sugar intakes. In a European review [31], added sugars were mainly contributed by sweet products (40 to 50%), followed by beverages (20 to 34%, excluding fruit juices) and then by dairy products (6 to 18%). In contrast, in the US, for children aged <2 years old [36], top sources of added sugars were fruit drinks (19.6%), sweet bakery products (14.9%) and sugar and candy (10.3%), with yogurt at 9.0% and dairy drinks (flavored milk and milk substitutes) at 5.6%.

### 4.3. Strengths and Limitations

The assessment of a representative sample of the Spanish children aged one to <10 years living in urban areas and the comparison of this reference population with a sample of adapted milk consumers of the same age segment is one of the main strengths of the EsNuPI study. Special care was taken in the design, protocol, and methodology of the study to ensure a correct sampling procedure. However, the fact that children living in rural areas were not included could be considered a potential limitation, although 52.6% of the Spanish population aged 1 to <10 years lives in urban areas [20]. Furthermore, although the 24-h DR information was collected following the methodology recommended by EFSA (The PAN CAKE-Pilot study) [40], we cannot fail to acknowledge the potential recall bias introduced by parents in reporting their children’s diets, which is associated with a tendency to overestimate foods accepted as healthy and underestimate the least healthy options. However, the assessment of misreporting from the EsNuPI study performed by Madrigal et al. [24] showed that the exclusion of under or over reporters has no significant influence in children’s TEI.

Finally, the fact that the European Regulation for the labeling of food products [28] does not require the quantification and declaration of added sugars on the labels—where only “carbohydrates” and “of which sugars” can be found, with this “sugar” content contemplating both intrinsic and extrinsic quantities—is an obvious limitation for any study that intends to assess these intakes, as well as for the elaboration of updated food composition tables and databases that distinguish between the different types of sugars.

## 5. Conclusions

Median estimates of intrinsic and added sugar intakes in the Spanish EsNuPI population as well as adherence to available recommendations varied notably according to age segment, but not substantially across milk consumer samples as children consuming adapted milks did not show a higher sugar intake than those from the reference sample population. Main food sources of intrinsic sugars for both samples were milk and dairy products, fruits, vegetables and cereals, while for added sugars, these were milk and dairy products (mainly yogurts), sugars and sweets, bakery products and cereals. Of interest, in the sample of adapted milk consumers, the groups milk and dairy products and cereals showed a significantly lower contribution to intrinsic sugar intake but a significantly higher contribution to that of added sugars.

Taking into account that younger children showed the highest added sugar contribution to TEI, and that in older children only half of them achieved intakes lower than the recommended 10% contribution to TEI, it is evident that education in nutrition, intervention measures and food policies encouraging product reformulation should be a priority targeting the younger population.

The urgent need of making efforts to facilitate the assessment of sugar intake is also evident. This involves harmonizing the criteria of the recommendations and the study methodology referring to either free or added sugars, as well as the declaration of their content in the nutritional labeling of products in order to include them in food composition databases.

## Figures and Tables

**Table 1:** Usual intrinsic and added sugar intakes by gender and age group in the Spanish Pediatric Population (EsNuPI) study.

	Group	Age Group	Boys		Girls	
			*n*	Median (P25–P75)	*n*	Median (P25–P75)
Intrinsic Sugars (g/day)	SRS	1 to <3 years	84	64.45 (55.1–81.2)	78	64.9 (48.5–81.8)
		3 to <6 years	122	64.8 (49.6–77.2)	122	65.8 (52.3–81.5)
		6 to <10 years	151	67.4 (52.8–82.4)	150	60.7 (47.6–71.9)
	AMS	1 to <3 years	144	59.4 (48.1–79.6)	150	56.5 (48.7–72.0) *
		3 to <6 years	128	64.4 (53.3–77.4)	134	63.1 (51.4–76.7)
		6 to <10 years	99	67.5 (49.6–81.8)	86	67.2 (45.7–79.6)
Added Sugars (g/day)	SRS	1 to <3 years	84	37.0 (27.8–47.3)	78	36.9 (26.0–42.1)^,^
		3 to <6 years	122	37.4 (29.2–46.6)	122	37.2 (28.6–45.7)
		6 to <10 years	151	41.4 (28.8–52.8)	150	35.9 (26.1–46.7)
	AMS	1 to <3 years	144	33.8 (26.0–46.1) ^a^	150	32.7 (26.1–40.7) *^,a^
		3 to <6 years	128	38.6 (30.1–48.3) ^a,b^	134	38.6 (29.8–46.7) ^b^
		6 to <10 years	99	40.1 (31.2–51.4) ^b^	86	42.9 (28.4–53.3) ^b^

Spanish Reference Sample (SRS) and Adapted Milk Consumers Sample (AMS). Values are presented as median (interquartile range). Different superscript lowercase letters (a and b) indicate statistical significance between age groups for each gender and in each sample type (*p* ≤ 0.05; Kruskal–Wallis test and the Dunn test to adjust for multiple comparison and adjust the *p*-value with Bonferroni correction) and asterisk indicates statistically significant difference between sample type and reference sample (*p* ≤ 0.05; Mann–Whitney’s U test).

**Table 2:** Added sugar distribution as a percentage of total energy (%) in the Spanish Pediatric Population (EsNuPI) study.

	Group	Age Group	Boys		Girls	
			*n*	Median (P25–P75)	*n*	Median (P25–P75)
Added sugars (%)	SRS	1 to <3 years	84	12.5 (9.0–15.4) ^a^	78	11.7 (9.1–15.3) ^a^
		3 to <6 years	122	10.1 (7.8–12.6) ^b^	122	10.1 (8.6–12.1) ^a,b^
		6 to <10 years	151	10.1 (7.0–12.1) ^b^	150	9.4 (7.2–11.9) ^b^
	AMS	1 to <3 years	144	12.2 (9.3–15.7) ^a^	150	11.3 (8.9–14.8) ^a^
		3 to <6 years	128	11.0 (8.3–13.0) ^b^	134	10.3 (8.6–12.3) ^b^
		6 to <10 years	99	9.8 (7.9–12.5) ^b^	86	11.0 (8.1–12.9) *^,a,b^

Spanish Reference Sample (SRS) and Adapted Milk Consumers Sample (AMS). Values are presented as median (interquartile range). Different superscript lowercase letters (a and b) indicate statistical significance between age groups for each gender and in each sample type (*p* ≤ 0.05; Kruskal–Wallis test and the Dunn test to adjust for multiple comparison and adjust the *p*-value with Bonferroni correction) and * *p* ≤ 0.05 difference between sample type and reference sample (Mann–Whitney’s U test).

**Table 3:** Prevalence of adequacy (percentage of population below 10% total energy intake) in the Spanish Pediatric Population (EsNuPI) study.

	Group	Age Group	Boys		Girls	
			*n*	% <10% Total Energy Intake	*n*	% <10% Total Energy Intake
Added sugars (%)	SRS	1 to <3 years	84	27.4	78	37.2
		3 to <6 years	122	49.2	122	46.7
		6 to <10 years	151	49.7	150	58.0
	AMS	1 to <3 years	144	31.3	150	34.7
		3 to <6 years	128	40.6	134	44.8
		6 to <10 years	99	53.5	86	39.5 *

Spanish Reference Sample (SRS) and Adapted Milk Consumers Sample (AMS). Values are presented as percentage. * *p* ≤ 0.05 difference between sample type and reference sample (Mann–Whitney’s U test).

**Table 4:** Prevalence of adequacy (percentage of population below 5% total energy intake) in the Spanish Pediatric Population (EsNuPI) study.

	Group	Age Group	Boys		Girls	
			*n*	% <5% Total Energy Intake	*n*	% <5% Total Energy Intake
Added sugars (%)	SRS	1 to <3 years	84	0.0	78	2.6
		3 to <6 years	122	6.6	122	3.3
		6 to <10 years	151	6.0	150	7.3
	AMS	1 to <3 years	144	1.4	150	3.3
		3 to <6 years	128	4.7	134	1.5
		6 to <10 years	99	6.1	86	2.3

Spanish Reference Sample (SRS) and Adapted Milk Consumers Sample (AMS). Values are presented as percentage.

**Table 5:** Added sugars usual intakes by age group and body composition in the Spanish Pediatric Population (EsNuPI) study.

	Group	Age Group	Underweight (%)		Normal Weight (%)		Overweight (%)		Obesity (%)	
			*n*	Median (P25–P75)	*n*	Median (P25–P75)	*n*	Median (P25–P75)	*n*	Median (P25–P75)
Added Sugars (g/day)	SRS	1 to <3 years	51	36.9 (27.6–45.6)	153	37.2 (28.3–43.1)	30	37.2 (25.4–48.8)	28	39.7 (27.9–46.1)
		3 to <6 years	27	32.3 (22.9–46.8) *	131	35.3 (28.2–49.0)	36	41.8 (30.4–55.2)	17	39.8 (24.5–49.5)
		6 to <10 years	41	35.0 (24.7–47.3)	149	39.6 (28.4–50.5)	40	37.5 (29.2–59.3)	4	30.0 (11.1–58.4)
	AMS	1 to <3 years	69	34.1 (27.6– 42.8)	234	34.8 (26.8–46.2)	68	34.3 (26.5–42.9)	43	34.0 (29.1–41.6)
		3 to <6 years	36	41.2 (33.0–56.1)	116	38.7 (30.6–50.6)	22	39.3 (31.8–49.4)	12	40.5 (28.8–45.2)
		6 to <10 years	23	41.8 (23.9–48.5)	89	42.8 (30.5–52.2)	27	36.7 (27.5–51.6)	2	34.5 (25.4–no data)

Spanish Reference Sample (SRS) and Adapted Milk Consumers Sample (AMS). Values are presented as median (interquartile range). * statistically significant difference between sample type and reference sample (*p* ≤ 0.05; Mann–Whitney’s U test).

**Table 6:** Usual added sugar intakes by income level and age groups from the Spanish Pediatric Population (EsNuPI) study.

	Group	Age Group	DK/NO		<600€ Per Month		600–1000€ Per Month		1001–1500€ Per Month		1501–2000€ Per Month		2001–3000€ Per Month		3001–4000€ Per Month		>4000€ Per Month	
			*n*	Median (P25–P75)	*n*	Median (P25–P75)	*n*	Median (P25–P75)	*n*	Median (P25–P75)	*n*	Median (P25–P75)	*n*	Median (P25–P75)	*n*	Median (P25–P75)	*n*	Median (P25–P75)
Added Sugars (g/day)	SRS	1 to <3 years	68	38.7 (28.2–46.5)	11	35.7 (23.2–39.4)	17	33.5 (28.6–44.4)	43	37.5 (26.2–43.9)	49	36.2 (27.5–46.0)	47	38.7 (29.6–44.0)	25	33.2 (25.3–43.7)	2	36.1 (34.5–no data)
		3 to <6 years	57	35.3 (25.3–48.0)	6	38.3 (21.8–55.1)	12	42.1 (21.4–59.6)	27	40.7 (32.7–57.2)	34	40.4 (30.1–51.0)	55	31.0 * (24.4–48.3)	17	38.6 (24.2–45.4)	3	35.0 (27.2–no data)
		6 to <10 years	59	34.6 (24.1–45.9)	13	42.8 (35.2–53.1)	10	38.0 (35.8–43.5)	32	30.3 (27.4–39.3)	43	41.3 (30.0–56.2)	58	43.2 (28.8–55.8)	17	41.4 (32.4–53.3)	2	42.0 (23.1–no data)
	AMS	1 to <3 years	111	35.2 (28.7–47.3)	8	34.5 (26.6–45.6)	28	31.0 (26.1–45.7)	45	33.4 (25.8–41.4)	88	33.5 (26.6–44.3)	96	34.1 (26.9–46.2	36	31.1 (24.6–42.1)	2	42.2 (29.5–no data)
		3 to <6 years	61	38.7 (32.5–51.9)	6	39.8 (35.6–62.6)	13	42.7 (30.8–49.5)	23	33.1 (26.3–44.7)	21	43.4 (25.5–57.7)	43	40.6 (33.3–53.1)	17	41.6 (38.6–47.3)	2	36.0 (24.0–no data)
		6 to <10 years	34	43.1 (30.5–52.2)	2	61.3 (38.2–no data)	11	40.1 (20.5–64.8)	27	32.9 (25.4–44.6)	25	43.4 (33.8–50.8)	34	43.1 (33.9–52.8)	6	52.5 (21.1–62.1)	2	30.8 (23.3–no data)

Spanish Reference Sample (SRS), Adapted Milk Consumers Sample (AMS) and do not know/no opinion (DK/NO). Values are presented as median (interquartile range). * statistically significant difference between sample type and reference sample (*p* ≤ 0.05; Mann–Whitney’s U test).

## Data Availability

The data presented in this study are available on request from the corresponding author.

## Supplementary Materials

The following are available online at https://www.mdpi.com/article/10.3390/nu14081667/s1, Table S1: Main food groups and subgroups used in the EsNuPI study.
