# Supplementary material for: Dietary Intake of Individual (Intrinsic and Added) Sugars and Food Sources from Spanish Children Aged One to <10 Years—Results from the EsNuPI Study
Source: Nutrients. 2022 Apr 16;14(8):1667. doi: 10.3390/nu14081667 (PMC9024444; doi:10.3390/nu14081667)
Supplement: Supplementary file 1 [file nutrients-14-01667-s001.zip › nutrients-1632761-supplementary.pdf]

Table S1. Main food groups and subgroups used in the EsNuPI study.

Group	Subgroups
**Cereals and derivatives**	Bread<br>Breakfast cereals<br>Rice and pasta
**Milk and dairy products**	Fresh cheese and cured cheese<br>Milk: natural, standard (cow's, goat's…)<br>Milk: adapted formulas (infant, follow-on, toddler's, enriched, fortified)<br>Yogurt and kefir
**Other dairy products**	Condensed milk and cream<br>Milk shakes and flavoured milk<br>Sweetened dairy desserts and ice cream
**Eggs**	Eggs
**Sugars and sweets**	Chocolate and confectionery<br>Sugar, honey, and marmalade<br>Sugary cocoa, nougat
**Oils and fats**	Margarine and butter<br>Olive oil<br>Other vegetable oils and lard
**Vegetables and potatoes**	All kind of vegetables<br>Potatoes
**Legumes**	Legumes
**Fruits**	All kinds of fresh fruits<br>Dried fruits<br>Fruits with juice and fruits with syrup<br>Olives
**Nuts**	Nuts
**Meat and meat products**	"Serrano" ham, cooked ham, sausages, other processed meats<br>Chicken and turkey with or without skin, medium-fat meat, fatty pork,<br>Lamb meat, rabbit meat, or liver and other viscera
**Fish and shellfish**	White fish, bluefish (small and large), fresh seafood, and canned fish
**Beverages**	Low-calorie carbonated beverages<br>Mixtures of fruit juice and milk<br>Natural juices and commercial juices<br>Sugar-sweetened carbonated beverages
**Sauces and condiments**	Sauces and Condiments

**Ready to cook/eat**	Homemade pre-cooked products<br>Industrial pre-cooked products
**Appetizers**	Snacks (fried snacks)
**Cereal-based baby foods and supplements**	
**Bakery products (Bakery and pastry)**	Cookies and cakes<br>Homemade bakery products<br>Shortbread cookies
